# Tobacco consumption in Spain: Individual risk profiles

**DOI:** 10.18332/tid/175044

**Published:** 2023-12-12

**Authors:** Imanol L. Nieto-Gonzalez, Carolina M. Rodriguez-Donate, Ginés Guirao-Pérez

**Affiliations:** 1 Universidad de La Laguna, San Cristóbal de La Laguna, Spain

**Keywords:** health, prevention, lifestyle, multinomial logit, sociodemographic factors

## Abstract

**INTRODUCTION:**

The aim of this study is to identify profiles of never smokers, ex-smokers, occasional smokers, and daily smokers, through their individual, lifestyle, and health characteristics. This analysis allows provides profiles of individuals with a greater or less propensity to use tobacco, which contributes to the design of effective prevention policies.

**METHODS:**

Econometric models are used with data from the Spanish National Health Survey. Specifically, a multinomial logit model is estimated to evaluate the probabilities of tobacco use. Additionally, discrete changes, odds ratios, and predicted probabilities for prone individuals, are calculated.

**RESULTS:**

Differences are found between the profiles of each alternative of tobacco use. The individual attributes with the most striking effect are being aged 26–45 years, which reduces the probability of being a non-smoker by 21 percentage points compared to the younger group, and regular exposure to secondhand smoke, which is 30% more likely to be a regular smoker. The characteristics that define an individual with higher probability of smoking daily are: belonging to a certain region of Spain, male, aged 26–45 years, born in Spain, unemployed, with primary studies, separated, no physical activity, consumes alcohol, and is exposed to smoke regularly, not chronically ill, and have very poor health.

**CONCLUSIONS:**

Identification of the profiles most likely to choose each of the tobacco consumption alternatives can contribute to the design of more effective prevention strategies. The results confirm that the accumulation of bad habits results in a high risk of smoking. The quantification of the differences in the effects of each trait is an interesting contribution that is useful to orient the policies to specific segments of the population more prone to consume tobacco.

## INTRODUCTION

Despite the efforts made by the various health authorities, the use of substances harmful to health and the addiction that they often generate, remain two major public health problems. Of these substances, alcohol and tobacco use among the general population stands out, although the use of other substances, in particular hypnotics and illegal drugs, is also a cause for concern. According to the Spanish Observatory of Drugs and Addictions^1^, more than 60% of Spanish adults aged 15–64 years, have consumed alcohol in the last 30 days, the same period in which 15.4% have done so excessively, especially among men aged 15–34 years. Furthermore, according to this report, almost 4 out of 10 Spaniards have used tobacco in the last year, being a regular practice for more than 30% of the population.

Tobacco use causes the death of more than eight million people worldwide each year^2^, of whom more than 1.2 million did not even use tobacco. This makes tobacco use the leading cause of preventable death in developed countries^3,4^. Nationally, annual tobacco-related deaths in Spain are estimated at more than 52000 people^5^. However, the consequences of smoking are not only, as mentioned above, a major problem from a health perspective, but also represent a huge economic and social cost, whose negative externalities are not alleviated through, for example, tax revenues from excise duties.

**Figure 1:**
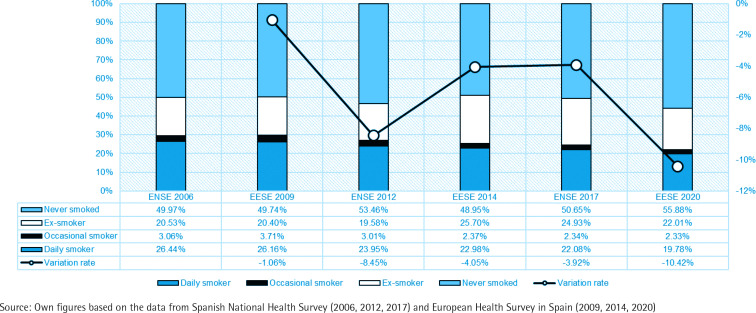
Evolution of the number of daily and occasional smokers, ex-smokers and never smokers in Spain 2006–2020 (% of total population)

In Spain, the percentage of smokers is almost two percentage points above the European average, which stands at 18.4%, despite the fact that the percentage of daily smokers has not stopped decreasing, at year-on-year rates ranging from 1% to 10% from 2009 to 2020 (Figure 1). These data highlight the need to insist on preventive policies^6^.

In recent years, preventive legislation on tobacco has been passed (Law 28/2005, 26 December; Law 42/2010, 30 December), which aims to reduce tobacco consumption, including bans on the use of tobacco in public spaces. In this respect, several previous studies have analyzed the effects of the entry into force of these laws and conclude that the effect of these laws has not been as expected^7-10^. On the other hand, other studies^11^ show that after the application of the law, cigarettes were considered as a less attractive and accessible product. Furthermore, research has pointed out that these laws have different levels of impact depending on the social class of the population. However, these legal frameworks that aim to significantly discourage smoking, are not enough^12^. In the same context, taxation should also be considered as a way of limiting tobacco consumption because of its direct effect on individuals’ purchasing power^13^.

Consequently, it is necessary to implement smoking prevention strategies^14^, beyond the enactment of legal precepts. However, although it is essential to evaluate the effectiveness of these policies, in many cases, such evaluation is not carried out, which reduces their measured effectiveness^15^. The lack of success of some preventive campaigns lies in their generality, without considering the individual differences of groups, or identifying relationships between the characteristics of the individuals and the habits to be treated^16^.

In this context, the aim of this study is to identify the impact of individual, lifestyle, and health characteristics on the probability of tobacco use in Spain. This analysis provides profiles of individuals with a greater or less likelihood to use tobacco, which may contribute to the design of effective prevention policies.

## METHODS

### Study design and setting

The data used in this study come from the Spanish National Health Survey for 2017, conducted by National Statistics Institute and within an appropriate ethical framework^17^. The survey provides information on the state of health of the population, its determinants and accessibility to health services, among other issues. Its objective, therefore, is to monitor the health of the population resident in Spain, compiling demographic, socioeconomic and territorial characteristics, including representative data on a national and regional level. It is carried out periodically, every five years, by means of a personal interview and is made up of 3 questionnaires: a household questionnaire, an adult questionnaire (age ≥15 years) and a questionnaire for minors (age <15 years). The original sample consisted of 23089 individuals. Sixty of them were eliminated due to their lack of response to some variables of interest in the analysis, so the final sample used was 23029 individuals.

### Measures

The microdata used in this study come from the Spanish National Health Survey adult questionnaire (age ≥15 years) and include the information on smoking decisions, their sociodemographic traits, as well as data concerning life habits and health determinants modules. We have considered a first group of variables that can be classified as biological or sociodemographic, such as gender, age, education level, and family situation, among others, which are usually related to the health status of the individual and are widely used in the literature. Moreover, empirical evidence indicates that these variables consistently contribute to explaining the decision to smoke. On the other hand, a second group of variables related to lifestyle habits, among which physical activity and alcohol consumption were selected to modulate the probability of tobacco consumption insofar as they may or may not act as facilitators of the acquisition of the habit. Finally, a third group of health variables, which may be taken as predisposing variables to certain alternatives, or which may further weigh the risk associated with tobacco consumption in that they differ in the effect that variables such as the presence of chronic disease and self-perceived health status have on tobacco consumption.

### Statistical analysis

To carry out the analysis, a discrete choice model is estimated, specifically, a multinomial logit model. The nature of the dependent variable of the model, as well as the type of data, will determine the most appropriate model. For the purpose of this study, given the discrete and unordered nature of the dependent variable, which presents more than two alternatives, the most appropriate specification is a multinomial model^18^. For more details on the methodology, see the Supplementary file.

Within the econometric model, the independence of irrelevant alternatives (IIA) was tested trough the Hausman specification test. The dependent variable of the model is the smoking decision and takes the values: 0=‘Never smoked’, 1=‘Ex-smoker’, 2=‘Occasional smoker’, and 3=‘Daily smoker’. The explanatory variables included in the model are sociodemographic characteristics of the respondent (autonomous region of residence, gender, age, origin, employment status, education level, and marital status), lifestyle habits (alcohol consumption, physical activity and exposure to indoor smoke) and health (chronic illness and self-perceived health status). For each of the traits considered, dichotomous variables have been defined that take the value 1 if the individual presents a specific modality in question and 0 otherwise. The modalities of each of the explanatory variables, as well as the reference modality of each one in the estimation of the model, can be found in the Supplementary file. All variables included in the model are significant at 99% confidence in at least one of the categories of the dependent variable. Furthermore, from the interpretation of the chi-squared test, it can be concluded that the variables are jointly significant at 1% significance, resulting in a usual value for McFadden’s R^2^.

## RESULTS

The sociodemographic characteristics of the respondents are shown in Table 1. The decision regarding tobacco consumption of the individuals surveyed shows an unequal distribution since 21.25% of the respondents smoke daily, 2.15% smoke occasionally, while 25.85% are ex-smokers, and 50.75% do not smoke and have never smoked regularly. When smoking status was analyzed according to the respondents’ sociodemographic characteristics (Table 2), it can be seen that, in relation to gender, the only category with a higher prevalence in females is that of non-smoker and the one with the highest in males is that of ex-smoker. In the group of occasional smokers, there is a greater weight of individuals aged 26–65 years, while within the category of non-smokers, it is those aged >65 years, who have a greater presence. In the case of ex-smokers and daily smokers, the modal range is 46–65 years old. In all consumption alternatives, individuals born in Spain have the highest proportion of smokers, although the lowest proportion of non-smokers or occasional smokers is noteworthy. The majority of smokers are employed, whereas in the group of never smokers or ex-smokers, the majority are inactive. The high percentage of individuals with primary education within the group of those who smoke on a daily basis, as well as those who have never smoked or are ex-smokers, stands out. In the case of occasional smokers, the majority have at least secondary studies. In all consumption alternatives there is a high percentage of married people, especially among ex-smokers (Table 2).

**Table 1:** Sociodemographic characteristics of the sample from the Spanish National Health Survey, 2017 (N=23029)

*Characteristics*	*n*	*%*
**Gender**		
Women	12463	54.12
Men	10566	45.88
**Age** (years)		
15–25	1796	7.80
26–45	6620	28.75
46–65	7944	64.50
>65	6669	28.96
**Employment status**		
Employed	9895	42.97
Unemployed	2481	10.77
Inactive	10653	46.26
**Education level**		
None	2741	11.90
Primary	9968	43.28
Secondary	4392	19.07
Higher	5928	25.74
**Marital status**		
Single	5883	25.55
Married	12452	54.07
Widowed	2969	12.89
Separated/divorced	1725	7.49
**Origin**		
Spain	20791	90.28
Foreign	2238	9.72
**Autonomous regions**		
Andalusia	2935	12.74
Aragon	1041	4.52
Asturias	836	3.63
Balearic Islands	915	3.97
Canary Islands	1115	4.84
Cantabria	790	3.43
Castilla y León	1282	5.57
Castilla La Mancha	1127	4.89
Catalonia	2348	10.20
Valencian Community	1831	7.95
Extremadura	951	4.13
Galicia	1332	5.78
Madrid	2027	8.80
Murcia	1025	4.45
Navarra	776	3.37
Basque Country	1494	6.49
La Rioja	669	2.91
Ceuta	255	1.11
Melilla	280	1.22

**Table 2:** Tobacco use by sociodemographic trait, lifestyle and health status of the sample from the Spanish National Health Survey, 2017 (N=23029)

	*Never smoker %*	*Ex-smoker %*	*Occasional smoker %*	*Daily smoker %*
**Total**	50.75	25.85	2.15	21.25
**Gender**				
Women	66.32	37.16	48.69	46.15
Men	33.68	62.84	51.31	53.85
**Age** (years)				
15–25	10.91	2.17	11.92	6.80
26–45	27.15	22.26	41.62	39.15
46–65	25.85	43.48	36.56	44.00
>65	36.09	32.09	9.90	10.05
**Origin**				
Spain	88.66	93.08	86.46	91.22
Foreign	11.34	6.92	13.54	8.78
**Employment status**				
Employed	36.56	44.86	58.99	54.34
Unemployed	8.27	8.82	12.73	18.92
Inactive	55.16	46.32	28.28	26.74
**Education level**				
None	15.98	9.93	3.64	5.39
Primary	41.86	42.57	34.95	48.40
Secondary	17.07	18.95	25.66	23.33
Higher	25.09	28.55	35.76	22.88
**Marital status**				
Single	26.44	17.00	38.18	32.52
Married	49.32	66.55	49.70	50.68
Widow	18.92	8.30	4.04	4.98
Separated/divorced	5.32	8.15	8.08	11.81
**Alcohol consumption**				
Not in the last 12 months	47.43	25.55	13.94	22.92
At least one day per month	27.97	26.14	33.33	29.48
At least one day per week	24.60	48.30	52.73	47.60
**Physical activity**				
Never	38.85	32.26	31.31	45.74
Occasionally	38.05	44.77	33.94	35.63
Monthly	10.86	11.91	16.36	9.91
Weekly	12.24	11.06	18.38	8.72
**Exposure to smoke**				
Never or rarely	93.75	93.72	87.27	69.60
Usually	6.25	6.28	12.73	30.40
**Chronic disease**				
Yes	69.42	75.87	60.20	61.78
No	30.58	24.13	39.80	38.22
**Health status**				
Very Good	19.53	14.81	22.02	18.49
Good	45.72	48.98	54.95	52.77
Fair	24.60	25.65	18.18	20.98
Poor	7.99	8.12	3.23	5.86
Very bad	2.16	2.44	1.62	1.90

Individuals who smoke or have smoked are those who, in a higher proportion, regularly consume alcohol. Moreover, those who have a higher frequency of tobacco consumption are the least physically active. Finally, in all categories of smoking status, most individuals are never or hardly ever exposed to smoke indoors. Though it is worth noting that more than 30% of daily smokers are regularly exposed to smoke, compared to <7% of non-smokers or ex-smokers.

In relation to the health variables, in all categories of the smoking status, the percentage of individuals who report having a chronic disease is the majority. This is especially notable in the case of ex-smokers, as >75% of them have a chronic disease. Regarding self-perceived health status in the last year, the most common health status is the same in all categories of tobacco consumption, with almost half of those who have never smoked or are ex-smokers considering their health status to be good, as well as more than half of those who smoke also affirming good health. In addition, in relative terms, fewer individuals who smoke consider their state of health to be very poor.

**Figure 2:**
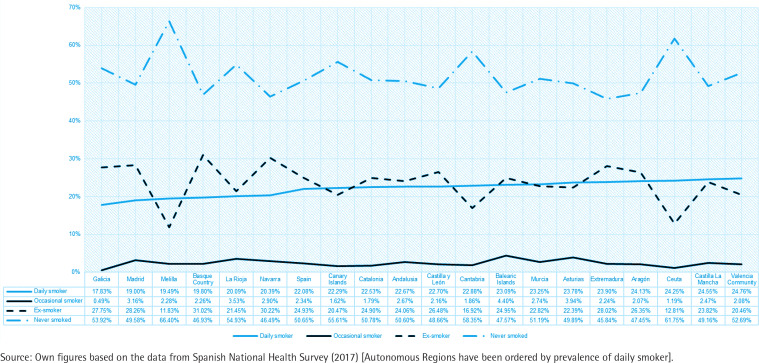
Evolution of the number of daily smokers in the autonomous regions of Spain 2017 (% of total population)

Finally, there are notable differences in tobacco consumption according to the Autonomous Community of residence (Figure 2). Based on these differences, two groups have been identified, differentiated according to the average percentage of non-smokers, depending on whether this is lower or higher than the national average. Thus, group 1 of Autonomous Communities presents an average percentage of 55%, while group 2 has an average percentage of non-smokers of 48% (Figure 2).

### Econometric model

The results of the multinomial logit model estimation are presented in Table 3. Individuals residing in the autonomous regions of the first group (CCAA_1) are more likely to be non-smokers than those residing in the autonomous regions of the second group. Men have a higher likelihood to be daily smokers and are also more likely to be ex-smokers. Younger individuals (reference category) are more likely to be non-smokers or occasional smokers than older individuals, however, as the age of the individual increases, the likelihood of quitting smoking increases and the likelihood of daily smoking decreases. Also striking is the negative sign of the discrete change in the alternative of higher consumption for older individuals, indicating that younger people are more likely to smoke daily than older people. This can be explained, among others, by two possible reasons: smokers die younger and the medical guidance not to smoke is more common in older individuals. Respondents born in Spain are 6.2% more likely to smoke daily than those born abroad. This result seems to be in line with the fact that the average number of smokers in Spain is above that of EU countries, however, they are also more likely to be ex-smokers. Individuals without formal education (reference category) were more likely to be non-smokers than other individuals, but they are also more likely to smoke on a daily basis than individuals with higher level of education. Individuals who are more likely to smoke daily are those with primary education. By contrast, those with secondary education are more likely to smoke occasionally, and finally, individuals with higher education are more likely to be ex-smokers. Employment status is also a discriminating factor in the decision to smoke. Inactive people are the most likely not to smoke or to have quit smoking. The fact that this group is made up of older people, mostly retired, together with the disincentive effect of age, could explain this result. Finally, single, married and widowed individuals are less likely to smoke on a daily basis than separated or divorced individuals, with the largest difference observed for widowers (9.2%).

**Table 3:** Estimates and discrete changes of the multinomial logit model, Spanish National Health Survey, 2017 (N=23029)

*Variable*	*Estimates*			*Discrete changes*			
	*Ex-smoker*	*Occasional smoker*	*Daily smoker*	*Never smoker*	*Ex-smoker*	*Occasional smoker*	*Daily smoker*
Constant	-3.626***	-4.760***	-3.870***	-	-	-	-
CCAA_1	-0.325***	-0.286***	-0.150***	0.05129	-0.04594	-0.00331	-0.00205
Men	1.035***	0.392***	0.621***	-0.17828	0.14860	-0.00110	0.03078
26–45 years	1.132***	0.329*	1.097***	-0.20814	0.09889	-0.00418	0.11343
46–65 years	1.705***	0.323	1.167***	-0.27433	0.19318	-0.00858	0.08972
>65 years	1.359***	-0.725***	-0.085	-0.13465	0.19640	-0.01913	-0.04262
Origin	0.423***	-0.024	0.640***	-0.09607	0.04119	-0.00671	0.06159
Employed	0.066	0.151	0.258***	-0.02931	-0.00240	0.00144	0.03028
Unemployed	0.199***	0.326*	0.710***	-0.08461	-0.00732	0.00159	0.09033
Primary	0.306***	0.430*	0.535***	-0.08199	0.02203	0.00371	0.05625
Secondary	0.409***	0.610**	0.502***	-0.09207	0.04047	0.00709	0.04451
Higher	0.290***	0.409	-0.017	-0.03707	0.04688	0.00628	-0.01609
Single	-0.492***	-0.065	-0.453***	0.09250	-0.05532	0.00468	-0.04187
Married	-0.179***	-0.349*	-0.687***	0.07954	0.01141	-0.00207	-0.08888
Widow	-0.809***	-0.715**	-0.937***	0.17504	-0.07901	-0.00460	-0.09143
No activity	0.028	0.015	0.857***	-0.07059	-0.03728	-0.00480	0.11267
Occasional activity	0.163***	-0.073	0.492***	-0.05337	0.00601	-0.00510	0.05246
Usually drink	0.870***	1.662***	1.103***	-0.21075	0.08772	0.02134	0.10169
Occasionally drink	0.492***	1.086***	0.559***	-0.11418	0.05458	0.01283	0.04677
Exposure usually	0.132*	0.565***	1.757***	-0.20070	-0.09946	-0.00236	0.30252
Chronic disease	0.225***	0.036	-0.110**	-0.01605	0.04293	0.00010	-0.02698
Good health	0.136***	0.256**	0.207***	-0.03410	0.01105	0.00336	0.01968
Fair health	0.276***	0.282*	0.260***	-0.05412	0.03177	0.00279	0.01957
Poor health	0.458***	0.058	0.348***	-0.08014	0.06061	-0.00280	0.02233
Very bad health	0.675***	0.830**	0.589***	-0.12981	0.08025	0.01112	0.03844

McFadden’s R^2^ =0.1505. Chi-squared test: χ^2^=7662.33 (0.00000). Log likelihood= -21629.95. Discrete changes have been calculated for each of the alternatives, for each individual in the sample and then averaged.

*** p <0.01;

** p<0.05;

* p<0.10.

In terms of an individual’s lifestyle habits, those who do not engage in any physical activity were the most likely to smoke on a daily basis, but at the same time, they are the least likely not to smoke or to quit smoking. Likewise, those who consume alcohol, either regularly or occasionally, or those who are frequently exposed to smoke indoors are also those who tend to smoke more frequently. In fact, individuals who are exposed to smoke at least one hour a day indoors were 30 percentage points more likely to smoke daily than those who are not exposed.

In relation to an individual’s state of health, it is found that individuals suffering from a chronic disease were 2.7% less likely to smoke daily than individuals who do not suffer from one, and 1.6% less likely to smoke at all. It is also observed that the worsening of self-perceived health status is associated with an increase in the probability of smoking. In addition, these individuals are more prone to smoke daily or quitting smoking too.

One of the most interesting contributions of this work is that it is possible to identify the profile of the individual most likely to choose each smoking status and to quantify the predicted probability. Next, based on the results obtained from the discrete changes, we present the modalities of each explanatory variable that define the profiles associated with the highest probability and include the predicted probability calculated from these profiles.

*Never smoker*

The profile of the individual more prone to be a never smoker, whose probability is 98.16%, is: CCAA_1, female, aged 15–25 years, not born in Spain, inactive, no studies, widowed, regular physical activity, no alcohol consumption, not exposed to smoke, not chronically ill, and in very good health.

*Ex-smoker*

The probability for ex-smoker is 71.96% and the characteristics are: CCAA_2, male, aged >65 years, born in Spain, inactive, higher studies, married, occasional physical activity, regular alcohol consumption, not exposed to smoke, chronically ill, and in very poor health.

*Occasional smoker*

In the case of occasional smoker (15.54%) the profile is: CCAA_2, female, aged 15–25 years, not born in Spain, unemployed, secondary studies, single, regular physical activity, regular alcohol consumption, not exposed to smoke, chronically ill, and in very poor health.

*Daily smoker*

The profile for daily smoker (91.85%) is: CCAA_2, male, aged 26–46 years, born in Spain, unemployed, primary studies, separated, no physical activity, regular alcohol consumption, regularly exposed to smoke, not chronically ill, and in very poor health.

**Figure 3:**
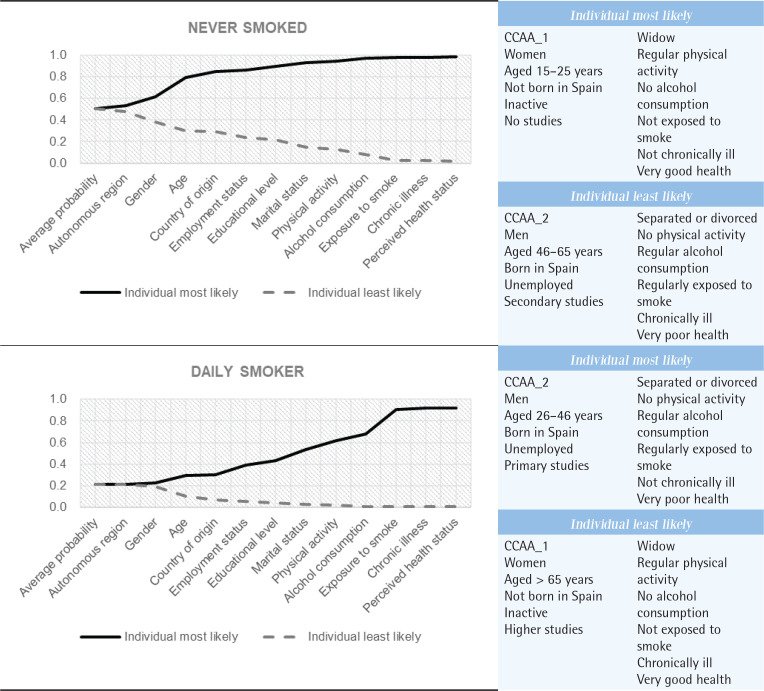
Evolution of the probability of the most and least prone individual from the Multinomial Logit Model, Spanish National Health Survey, 2017 (N=23029)

In addition, the cumulative nature of the different risks can be illustrated to predict the behavior of the two most extreme alternatives to tobacco consumption. Figure 3 shows, for the two extreme alternatives (never smoker and daily smoker), the evolution of the probability when incorporating each of the individual characteristics that make up the profile of the most and least prone individual, respectively, identified from the discrete changes calculated. From Figure 3, one can conclude the significant variations in terms of probability that the individual has one characteristic and not another. It is noted that the risk factors with the most significant influence on the likelihood of engaging in daily smoking are usual alcohol consumption, lack of physical activity, and frequent exposure to secondhand smoke. In contrast to socioeconomic and behavioral risk factors, the increased predictive capability of chronic illness and perceived health status is constrained. This limitation is evident in Figure 3, which shows minimal changes in the probability of identifying a daily smoker.

## DISCUSSION

There are multiple modifiable non-communicable disease risk factors such as unhealthy habits that are of global concern including tobacco use. This study explores the clustering of risk factors in the tobacco use. The results obtained agree with those reported in the literature^19^. The recent work by Martín et al.^20^, which, taking data from the 2014 National Health Survey for Spain, analyzes the factors that have a higher prevalence with cigarette smoking, and concludes that having no education, being unemployed, being male, divorced and having worse lifestyle habits, have a positive influence on tobacco consumption. Similar results to those obtained in the present study. Gallus et al.^21^ found a higher prevalence of smoking in men and among individuals aged 25–44 years. They also found an inverse relationship between smoking and higher education level. Gangani et al.^22^ coincide in pointing to a higher prevalence in men and middle-aged individuals and a decrease in smoking among the elderly. On the other hand, they report a positive association of tobacco use with higher alcohol consumption and lower education level. Diez-Gañán et al.^23^ highlight the association of less healthy lifestyle habits, such as less physical activity, with daily smoking as opposed to occasional smoking. Among the groups most prone to smoking, young people are the ones that most concern health authorities. Evidence suggests a high prevalence of non-communicable disease risk factors in adolescents and tobacco use is one of these^24^. Many studies point to the need to implement prevention plans in schools^25-26^, as well as banning smoking in places frequented by children^27^. There are several reasons for young people’s propensity to use tobacco and alcohol^28^, but the common denominator is the social and family environment, which is often the basis for the initiation of tobacco use^29^. Social acceptance and stress relief contribute to the maintenance of smoking among this segment of the population^30^. Kelly et al.^31^ consider that denormalization is a key mechanism that affects smoking among young people. Personal or sociodemographic aspects are also associated with smoking initiation^32-35^. Moreover, Sánchez et al.^36^ argue that the use of tobacco and other substances among young people is a rite of passage to adulthood. In this sense, Villalbí et al.^37^ point out the positive effect that discouraging tobacco use among adults can have on young people.

An additional aspect of interest is to the calculate odds ratios of two alternatives when compared to two individuals differentiated by some characteristic, which makes it possible to illustrate how the preference for one alternative changes after another. Respondents born in Spain are more likely to smoke daily compared to not smoking, to quit smoking or to quit smoking occasionally than those born outside Spain. It is also observed that the odds ratio decreases as education level increases, whatever the reference alternatives, indicating that the ratio representing the probability of smoking daily versus not smoking, decreases as the individual’s education level increases. In other words, the more educated the individual is, the lower the weight of frequent smoking compared to the other consumption alternatives. If an individual suffers from a chronic disease, the probability of smoking daily versus not smoking or quitting smoking or smoking sporadically lower, respectively, than that of an individual without a chronic disease.

Smoking is one of the lifestyle risk behaviors that increase disease burden globally, so targeted interventions are necessary^38^. Prevention plans or strategies are a key aspect of discouraging tobacco use. The implementation of price control policies or smoking restrictions in public places are among the most effective^39^. Although less successful, advertising controls, the inclusion of smoking cessation therapies in primary care, or health warnings on tobacco packages, are also control policies that have been implemented^40^. However, the empirical evidence on the effectiveness of such policies is not entirely robust, and they do not seem to discourage smoking initiation^40^. Villalbí et al.^37^, who analyze current tobacco control policies in Spain and the main innovations that should be included to improve their effectiveness, point out that the tobacco epidemic, as well as preventive policies, have a diverse reality.

### Limitations

Despite the significant findings of this study, several limitations should be considered. Firstly, the data used does not allow differentiating the profile of the consumer of electronic cigarettes, which has been increasing in recent years, compared to that of traditional tobacco. This increase is due to the erroneous healthy consideration for this kind of tobacco that it could move the demand to this modality despite that it is harmful too^41^. Secondly, given the self-reported nature of some variables, as alcohol consumption or physical activity, the effects could be different if they are collected in another form. Finally, it would be interesting doing a longitudinal analysis. As a future line of research, it would be interesting to identify smoking profiles among the young population and doing a longitudinal analysis.

## CONCLUSIONS

Tobacco prevention strategies should be targeted at population segments with particularly prevalent characteristics such as gender, country of origin, non-physical activity, alcohol consumption and time spent in enclosed places exposed to tobacco smoke. In this sense, the identification of the profiles most likely to choose each of the tobacco consumption alternatives can contribute to the design of these strategies.

## Data Availability

The data supporting this research are available from the following source: https://www.ine.es/dyngs/INEbase/es/operacion.?c=Estadistica_C&cid=1254736176783&menu=resultados&idp=1254735573175#!tabs-1254736195295
