# Supplementary material for: Tobacco consumption in Spain: Individual risk profiles
Source: Tob Induc Dis. 2023 Dec 12;21:164. doi: 10.18332/tid/175044 (PMC10714415; doi:10.18332/tid/175044)
Supplement: Supplementary file 1 [file TID-21-164-s1.pdf]

APPENDIX

METHODOLOGY (Further details)

To carry out the analysis, a discrete choice model is estimated, specifically, a multinomial logit model. In these models, based on the Random Utility Theory, developed by Domencich and McFadden (1975), each individual chooses from among a set of J alternatives, where each alternative A_j gives to individual i a certain utility U_{ij}, which is derived from the individual's own characteristics and from the attributes of the alternatives. This utility presents a systematic part (V_{ij}) and a random part (ε_{ij}), such that:

$$U_{ij} = V_{ij} + \varepsilon_{ij}$$

Individual i will choose alternative A_{j^*}, if and only if:

$$U_{ij^*} > U_{ij}, \forall j \neq j^*$$

That is to say:

$$V_{ij^*} + \varepsilon_{ij^*} > V_{ij} + \varepsilon_{ij}, \forall j \neq j^*$$

Thus, the probability that the individual chooses alternative A_{j^*}, can be expressed as:

$$P(Y_i = j^*) = P\left(U_{ij^*} > U_{ij}\right) = P\left(\varepsilon_{ij} - \varepsilon_{ij^*} < V_{ij^*} - V_{ij}\right) \quad \forall j \neq j^*$$

The nature of the dependent variable of the model, as well as the type of data, will determine the most appropriate model. Moreover, the distribution assumed for the vector of random disturbance terms ($\varepsilon_{i0}, \dots, \varepsilon_{iJ}$) will determine the functional form of the model. For the purpose of this paper, $P(Y_i = \text{j})$ refers to the probability of being non-smoker (j = 0), ex-smoker (j = 1), occasional smoker (j = 2) and daily smoker (j = 3), respectively. Given the discrete and unordered nature of the dependent variable, which presents more than two alternatives, the most appropriate specification is a multinomial model (Rodríguez Donate and Cáceres Hernández, 2007). Furthermore, a logistic function is assumed for the random disturbance terms, which implies the use of a multinomial logit model whose probabilities are expressed as follows:

$$P(Y_i = j) = \frac{e^{x_i' \beta_j}}{1 + \sum_{k=1}^{J} e^{x_i' \beta_k}} \quad j = 1, \dots, J$$

$$P(Y_i = 0) = \frac{1}{1 + \sum_{k=1}^{J} e^{x_i' \beta_k}} \quad j = 0$$

Where x_i is the vector of explanatory variables and β the set of parameters to be estimated. To interpret the results obtained, given that the parameters are not directly interpretable, it is useful to calculate marginal effects, if the explanatory variable is quantitative in nature, or to discrete changes, if it is qualitative.

The discrete change, i.e., the change in the probability of choosing alternative j when the categorical variable x_{im} changes from taking the value 0 to 1, is obtained from:

$$P(Y_i = j/x_{im} = 1) - P(Y_i = j/x_{im} = 0), j = 0, \dots, J$$

Also of interest is the calculation of the odds ratios, which show changes in the substitution pattern between alternatives when explanatory variables change. These are defined as:

$$\Omega_{j/k} = \frac{P(Y_i = j)}{P(Y_i = k)} = \frac{e^{x_i'\beta_j}}{e^{x_i'\beta_k}} = e^{x_i'(\beta_j - \beta_k)}, j = 0, \dots, J, j \neq k$$

The odds ratio when the explanatory variable x_{im} changes by one unit, i.e. $e^{(\beta_{jm} - \beta_{km})}, j = 1, \dots, J, j \neq k$, indicates the effect of such a change on the substitution pattern between alternatives j and k, while $e^{\beta_{jm}}$ measures the effect on the substitution pattern between alternative j and the reference alternative.

Domencich T, McFadden D. *Urban Travel Demand: A behavioural Analysis*. Amsterdam: North-Holland, 1975.

Rodríguez Donate MC, Cáceres Hernández JJ. Modelos de elección discreta y especificaciones ordenadas: una reflexión metodológica. *Estadística Española* 2007; 49 (166): 451-471.

Table A.1. Variables included in the model

VARIABLE	DESCRIPTION	VALUES
	SOCIO-DEMOGRAPHIC	
CCAA_1	Autonomous Regions of residence	1 = CCAA_1 (Andalusia, Asturias, Canary Islands, Cantabria, Catalonia, Valencian Community, Galicia, La Rioja, Ceuta, Melilla)
CCAA_2		1 = CCAA_2* (Aragón, Balearic Islands, Castilla La Mancha, Castilla y León, Extremadura, Madrid, Murcia, Navarra, Basque Country)
MEN	Gender	1 = Men
WOMEN		1 = Women*
AGE	A1	1= 15-25 years old*
	A2	1= 26-45 years old
	A3	1= 46-65 years old
	A4	1= more than 65 years old
ORIGIN	Country of origin	1 = Spain; 0=Foreign*
EMP	Employment status	1 = Employee
UNEMP		1 = Unemployed
INACT		1 = Inactive*
NO_ST*	Educational level	1 = No studies*
PR_ST		1 = Primary studies
SE_ST		1 = Secondary studies
HIG_ST		1 = Higher studies
SINGLE	Marital status	1 = Single
MARRIED		1 = Married
WIDOW		1 = Widow
SEP_DIV		1 = Separated or divorced*
	LIFESTYLE HABITS	
NO_FA	How often do they do any physical activity in their free time?	1 = Never
OCCAS_FA		1 = Occasional
USUAL_FA		1 = Usually* (Monthly/Weekly)
USUAL_ALC	Frequency of alcohol consumption in the last 12 months	1 = At least one day a week
OCCAS_ALC		1 = At least one day per month
NO_ALC		1 = Not in the last 12 months*
EXPT_NO	Frequency of exposure to smoke indoor environments	1 = Never or hardly ever*
EXPT_USUAL		1 = At least one hour a day (Usually)
	HEALTH	
CHRONIC	Chronic or long-term illness or health problem	1 = Yes; 0= No*
HS_VG	Perceived health status in the last 12 months	1 = Very good*
HS_G		1 = Good
HS_G		1 = Regular
HS_B		1 = Bad
HS_VB		1 = Really bad

* Reference category in the model.

© 2023 Imanol L. Nieto-González et al.
